# Research on Radiation-Hardened RCC Isolated Power Supply for High-Radiation-Field Applications

**DOI:** 10.3390/mi16101135

**Published:** 2025-09-30

**Authors:** Xiaojin Lu, Hong Yin, Youran Wu, Lihong Zhu, Ke Hong, Qifeng He, Ziyu Zhou, Gang Dong

**Affiliations:** 1 School of Integrated Circuits, Xidian University, Xi’an 710000, China; luxiaojincetc@163.com (X.L.);; 2 The 43rd Research Institute of China Electronics Technology Group Corporation, Hefei 230088, China; 3 National Key Laboratory of Vacuum Technology and Physics, Lanzhou Institute of Physics, Lanzhou 730000, China

**Keywords:** nuclear environment, auxiliary power supply, displacement damage effect, VDMOS

## Abstract

A radiation-hardened RCC (Ring Choke Converter) isolated power supply design is proposed, which provides an innovative solution to the challenge of providing stable power to the PWM controller in DC-DC converters under nuclear radiation environments. By optimizing circuit architecture and component selection, and incorporating transformer isolation and dynamic parameter compensation technology, the RCC maintains an 8.9 V output voltage after exposure to neutron irradiation of 3 × 10^13^ n/cm^2^, significantly outperforming conventional designs with a failure threshold of 1 × 10^13^ n/cm^2^. For the first time, the degradation mechanisms of VDMOS devices under neutron irradiation during switching operations are systematically revealed: a 32–36% reduction in threshold voltage (with the main power transistor dropping from 5 V to 3.4 V) and an increase in on-resistance. Based on these findings, a selection criterion for power transistors is established, enabling the power supply to achieve a 2 W output in extreme environments such as nuclear power plant monitoring and satellite systems. The results provide a comprehensive solution for radiation-hardened power electronics systems, covering device characteristic analysis to circuit optimization, with significant engineering application value.

## 1. Introduction

In military electronics applications such as aerospace and aviation, various subsystems—including constant current sources, DC-DC converters, and servo control systems—require auxiliary power supplies to maintain stable operation. These systems often operate in radiation environments, one of which is nuclear radiation. Nuclear radiation environments can be classified into two categories: nuclear explosion environments and nuclear power environments. The former refers to the space where a large number of high-energy particles and intense electromagnetic pulses are generated during a nuclear explosion. The latter involves continuous radiation generated by nuclear-powered facilities such as nuclear power plants, nuclear submarines, and other platforms equipped with nuclear energy sources. In such environments, the main high-energy particles include fast neutron fluxes, high-energy electron beams, gamma rays (γ-rays), x-rays, alpha particles (α-rays), and beta particles (β-rays) [1,2]. Exposure to nuclear radiation alters the electrical characteristics of semiconductor devices due to the displacement damage effect (DDE). This effect is caused by incident radiation particles colliding with atoms in the semiconductor lattice, displacing them from their original positions and forming lattice defects such as interstitial atoms and vacancies. A vacancy and its adjacent interstitial atom are known as a Frenkel pair, while two adjacent vacancies induced by radiation are referred to as a divacancy [3,4,5]. Displacement damage can result in output voltage drift or even complete failure of auxiliary power circuits or modules, making them incapable of sustaining normal operating conditions. Conventional semiconductor devices are primarily based on silicon (Si) and fall into two categories: bipolar junction transistors (BJTs) and field-effect transistors (FETs). BJTs, being minority carrier devices, exhibit relatively strong tolerance to total ionizing dose (TID) effects from γ-rays but are highly sensitive to neutron irradiation. Key physical parameters affected by radiation include the base transit time and base width, which in turn degrade current gain, increase junction leakage current, raise saturation voltage, and reduce breakdown voltage [3], In conventional designs of DC-DC converters, bipolar devices are widely used, especially in auxiliary power supplies, and the neutron fluence threshold that bipolar devices can withstand is 1 × 10^13^ n/cm^2^ [6,7,8]. In contrast, FETs are majority carrier devices and respond differently to radiation. Neutron irradiation causes displacement effects that deplete charge carriers in the channel, impacting parameters such as transconductance, drain current, and pinch-off voltage. Meanwhile, γ-ray irradiation induces ionizing effects, where charge accumulation in the oxide layer at the Si/SiO_2_ interface introduces surface states, affecting gate leakage current and threshold voltage [9,10]. Comparatively, FETs demonstrate superior resilience to neutron-induced displacement damage compared to BJTs. Therefore, the design strategy adopted in this study emphasises minimising or eliminating the use of bipolar devices by replacing them entirely with radiation-tolerant FETs.

## 2. Circuit Schematic Block Diagram

**Figure 1:**
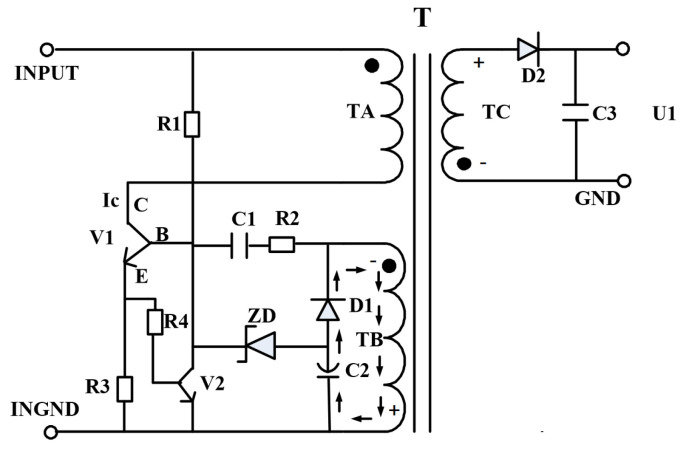
Schematic block diagram of a typical single-channel RCC circuit.

The Ring Choke Converter (RCC), also known as a self-excited flyback converter, features a simple structure and low cost. It operates through self-excited oscillation, where changes in external conditions—such as input voltage or output current—can result in significant variations in operating frequency (Figure 1). This leads to reduced power conversion efficiency, making the RCC unsuitable for high-power applications.

The operating principle is as follows: When the input voltage is applied, resistor *R1* provides a startup current to the switching transistor *V*, turning it on. The collector current *I_C_* through winding *T_A_* increases linearly, which induces a positive-top, negative-bottom electromotive force (EMF) in winding *T_B_*. This forward-bases the base-emitter junction of transistor *V* (base *B* positive, emitter *E* negative), quickly driving the transistor into saturation.

Simultaneously, the induced voltage charges capacitor *C1*. As the voltage across *C1* increases, the base voltage of transistor *V* gradually decreases, causing the transistor to exit saturation. The collector current *I*_*C*_ then begins to decrease, inducing an opposite EMF in winding *T_B_* (top negative, bottom positive), which reverses the base-emitter polarity (base *B* negative, emitter *E* positive) and forces the transistor to turn off rapidly.

When transistor *V* is turned off, no EMF is induced in *T_B_*, and the DC input voltage begins to reverse-charge capacitor *C1* via resistor *R1*. This gradually raises the base voltage of transistor *V*, initiating a new conduction cycle. The circuit thus enters continuous self-excited oscillation. The waveform at the base of transistor *V* is a square wave with a high level of approximately 0.7 V. The capacitance of *C1* determines the conduction time of the transistor, which in turn sets the switching frequency.

**Figure 2:**
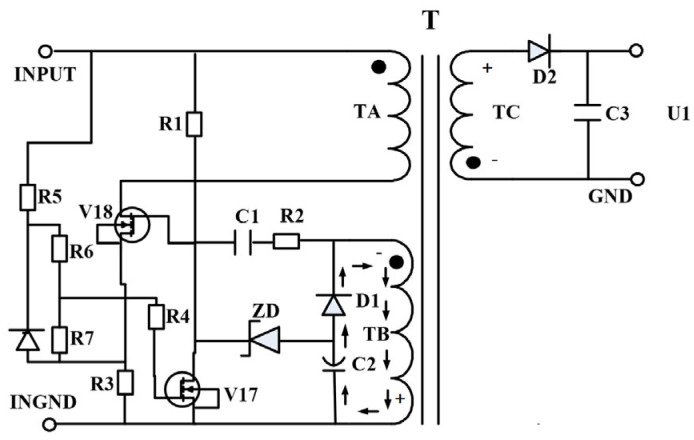
Optimized isolated auxiliary power supply schematic.

Based on the RCC’s self-excited oscillation principle, the circuit was optimized by replacing bipolar devices with field-effect transistors (FETs). A novel RCC-based auxiliary power circuit was developed, operating at a +12 V DC supply with approximately 2 W output power. The optimized circuit structure is shown in Figure 2.

As illustrated in Figure 2, the current-controlled BJT was replaced with a voltage-controlled Vertical Double-Diffused Metal-Oxide-Semiconductor (VDMOS) transistor. Both *V1* and *V2* VDMOS devices require a pre-driving gate-source (GS) voltage to operate correctly, enabling coordinated switching behavior. The remaining portions of the circuit remain functionally equivalent to the original BJT-based design.

## 3. Circuit Parameter Design

### 3.1. Selection of the Turns Ratio

As a boundary-conduction-mode (BCM) flyback converter, the RCC operates in a critical conduction regime. The relationship between the input and output voltage is defined by Equation (1):

(1) $$N(D)=\frac{D\times V_{Vin\_min}}{V_{0}\times (1-D)}$$

**Figure 3:**
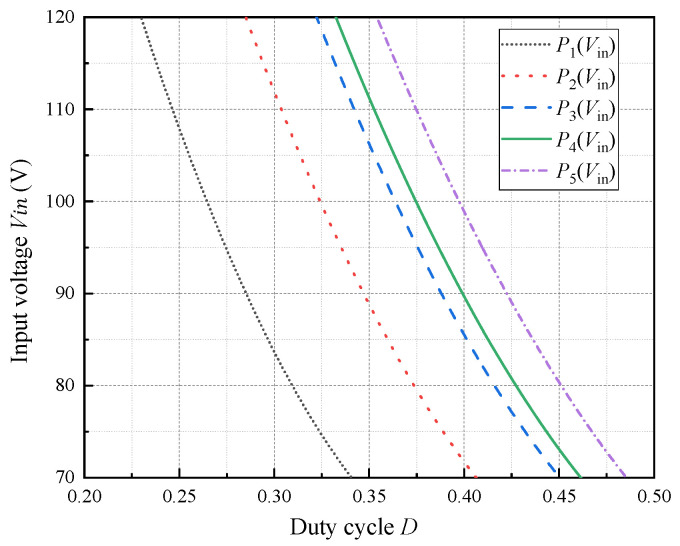
Relationship between input voltage *V*_*in*_ and duty cycle *D* for different transformer turns ratios *N*. Curves *P*_1_(*V*_in_), *P*_2_(*V*_in_), *P*_3_(*V*_in_), *P*_4_(*V*_in_), and *P*_5_(*V*_in_) correspond to *N* values of 3, 4, 4.77, 5 and 5.5, respectively.

At the minimum input voltage $V_{Vin\_min}$ = 70 V, the duty cycle *D* typically reaches its maximum, which generally does not exceed 0.45. Figure 3 illustrates how the duty cycle *D* varies with the input voltage *V_in_* under different transformer turns ratios *N*.

As illustrated in Figure 3, it can be seen that increasing the turns ratio shifts the curve to the right and increases the duty cycle. When *V*_*in(min)*_ = 70 V, the corresponding *Dmax* is approximately 0.45. A turns ratio of *N* = 4.77 is appropriate, which is rounded to an integer value of 5. The corresponding minimum duty cycle at the maximum input voltage is 0.33, resulting in a duty cycle range of 0.33–0.45, which is reasonable. Thus, *D_max_* = 0.462.

### 3.2. Frequency Selection

Based on the definition of oscillation frequency, the operating frequency *f* can be estimated by the following equation [11,12].

(2) $$f={\left(\frac{N\times V_{0}}{1+N\times \frac{V_{0}}{V_{in}}}\right)}^{2}\times \frac{1}{2P_{0}\times L_{P}}\times \eta$$

Here, *N* = 5, *V*_0_ = 12 V, *V*_in_ = 70~120 V, Output power *P*_0_ = 2 W, A fixed time constant *t* is assumed for simplification.

**Figure 4:**
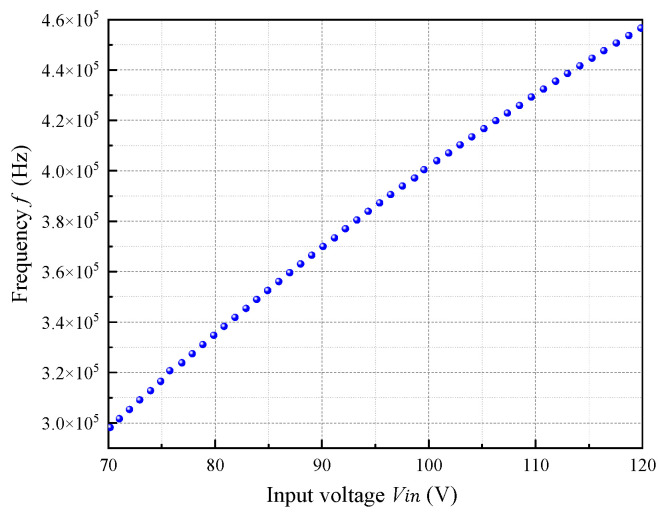
Frequency (*f*) versus input voltage (*V*_in_) under full-load conditions.

In RCC converters, the switching frequency is not strictly constant. Equation (2) shows that it dynamically varies based on input voltage, load conditions, and circuit parameters such as transformer inductance and feedback components [12]. Figure 4 illustrates the frequency versus input voltage relationship under full-load conditions, showing a positive correlation with a frequency range from 300 kHz to 460 kHz. For simplification in initial circuit design, a nominal frequency of 400 kHz is assumed, allowing estimation of inductance values, with the final output voltage adjusted by fine-tuning internal parameters.

### 3.3. Peak Primary Current *I*_*p*_

The primary peak current *I*_*p*_ is calculated as:

(3) $$I_{P}=\frac{2\times P_{0}}{\eta \times V_{in}\times D}$$

Here, the efficiency *η* is considered to be 60%, given the relatively low power level of the RCC circuit. At an output power P_0_ of 2 W, the efficiency does not exceed 65%; thus, a value of 60% is adopted. When the input voltage *V*_in_ reaches its minimum value of 70 V, the peak primary-side current reaches its maximum. Under these conditions, the corresponding duty cycle is 0.462, resulting in a maximum primary current *I*_p_max_ of 206 mA.

## 4. Transformer Design

The transformer uses ER7.5 series MnZn ferrite magnetic cores from Beijing Qixing Feihang. Key material parameters are:

Saturation flux density *B*_*s*_ = 3100 *G* (at 125 °C);

Remanent flu density *B*_*r*_ = 1200 *G* (at 125 °C);

Effective core area *A*_*e*_ = 5.8 mm^2^;

Core volume *V*_*e*_ = 64.3 mm^3^;

(1) Primary Turns *N*_*P*_

(4) $$N_{P}=\frac{V_{in\_\min}\times T_{on}}{A_{e}\times B_{s}}$$

Calculated as *N*_*P*_ = 44.97, rounded to 45 turns.

(2) Secondary Turns *N*_*S*_

(5) $$N_{s}=N_{p}\times \frac{V_{0}+0.2}{V_{in\_\min}}\times (\frac{1}{D_{\max}}-1)$$

Calculated as *N*_*S*_ = 9.13 N, rounded to 9 turns.

(3) Feedback Winding Turns *N*_*f*_

The feedback winding must induce a voltage greater than 5.5 V [13,14]:

(6) $$N_{f}=N_{p}\times \frac{5.5}{V_{in\_\min}}$$

Resulting in *N*_*f*_ = 3.54, rounded to 4 turns. Due to space limitations, other parameter details are not elaborated here.

## 5. Irradiation Test Results and Discussion

The RCC circuit serves as an isolated power supply for the PWM controller within a DC-DC converter, as highlighted in the red frame of the system schematic. Neutron irradiation experiments were conducted at the Northwest Institute of Nuclear Technology. A total neutron fluence of 3 × 10^13^ n/cm^2^ was applied while the DC-DC converter operated under room temperature and full-load conditions.

As illustrated in Table 1 and Table 2, it is evident that the converter remained functionally stable throughout the irradiation process, validating the feasibility of using the RCC circuit to provide stable power for PWM controllers. The most significant impact of neutron irradiation is observed in the converter’s efficiency. At the nominal input voltage of 100 V, efficiency dropped from 78.2% pre-irradiation to 77.5% post-irradiation. This degradation indicates changes in the electrical performance of semiconductor components, including VDMOS switches and Schottky rectifiers.

The following analysis focuses on the impact of irradiation, specifically on the RCC converter. As a low-power flyback converter with 2 W output, its performance pre- and post-irradiation is summarized in Table 3 and Table 4.

As illustrated in Table 3 and Table 4, it is observed that both output voltage and efficiency were significantly affected by irradiation. The output voltage decreased from 12.163 V to 8.981 V at nominal input, approaching the lower operating limit (8.8 V) of the PWM controller. When neutron fluence exceeds 3.2 × 10^13^ n/cm^2^, the RCC output falls below 8.8 V, rendering the DC-DC converter non-functional.

To maintain operability under higher irradiation doses, the pre-irradiation output voltage of the RCC must be increased, albeit at the expense of reduced efficiency. The decline in efficiency, which suggests an increase in the on-resistance of the VDMOS devices, results in higher conduction losses. Additionally, the growing deviations in voltage and load regulation indicate reduced circuit stability, implying that neutron-induced displacement damage significantly degrades the electrical characteristics of the VDMOS.

Subsequent sections will analyse in detail the degradation trends and performance impacts of VDMOS devices operating under neutron irradiation within the RCC circuit.

## 6. Results and Discussion

Table 5 shows the radiation-induced electrical characteristics of the N-type irradiated power VDMOS transistors selected for the RCC circuit: Drain-source breakdown voltage: *V*_(BR)DSS_; Gate-source threshold voltage: *V*_GS(th)_; Gate forward leakage current: *I*_GSSF_; Gate reverse leakage current: *I*_GSSR_; Off-state drain leakage current: *I*_DSS_; Drain-source on-resistance: *R*_DS(ON)_.

**Figure 5:**
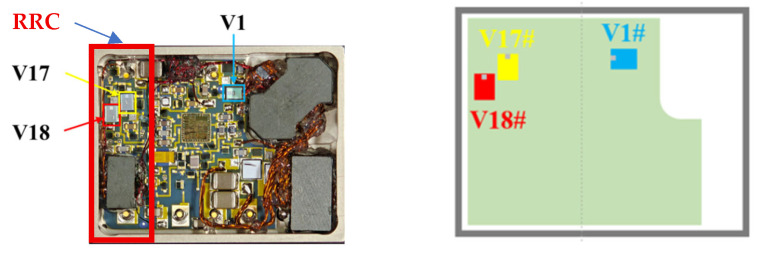
Layout diagram of VDMOS.

As shown in Figure 5, the power VDMOS positions in the DC/DC converter are illustrated. Here, *V1* is the switching transistor of the DC/DC converter, while *V17* and *V18* are the power VDMOS transistors in the RCC, where *V18* is the main switch and *V17* is the current-limiting transistor. Testing was conducted using the Semiconductor Parameter Analyzer 4200A-SCS, with three product samples containing internal VDMOS bare chips from the same batch of fabrication. The samples were tested under full load at room temperature, and the results were averaged.

**Figure 6:**
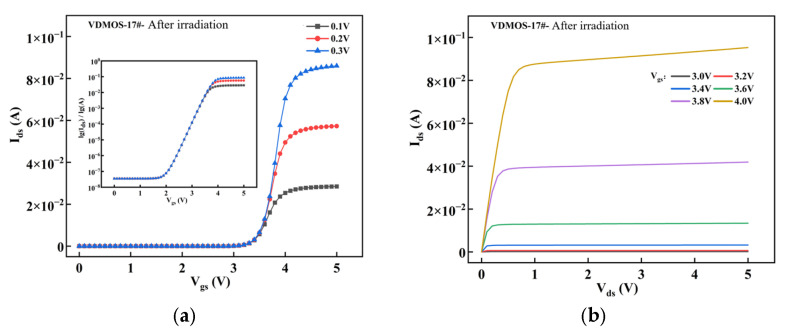

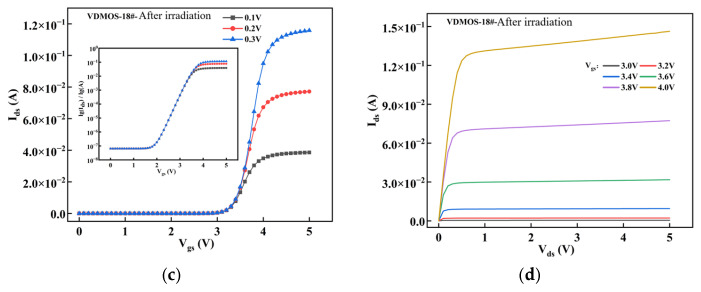
Displacement damage effects induced by neutron irradiation: (**a**) Transfer characteristics of *V17*, (**b**) Output characteristics of *V17*, (**c**) Transfer characteristics of *V18*, (**d**) Output characteristics of *V18*.

As shown in Figure 6, subfigures a and b represent the transfer and output characteristics of *V17*, while subfigures c and d represent those of *V18*. It can be observed that: after irradiation, the threshold voltage *V*_GS_(th) of *V17* shifts to 3.2 V, and the on-resistance *R*_DS_(ON) increases to 1.38 Ω, while for *V18*, *V*_GS_(th) shifts to 3.4 V and *R*_DS_(ON) increases to 1.25 Ω. Comparing *V17* and *V18*, both show a negative drift in threshold voltage, indicating the generation of significant oxide trap charges after irradiation. The threshold voltage of *V18* experiences a smaller negative drift compared to *V17*, suggesting that *V17* generates more oxide trap charges than *V18*. Furthermore, *V18* exhibits a lower on-resistance after irradiation compared to *V17*, with similar slope characteristics in both curves and a corresponding reduction in saturation drain current.

The observed phenomena suggest that for the switching transistors in the on-state, neutron irradiation caused displacement damage. Comparative Analysis of the Differences Between *V17* and *V18*: the extent of damage differs between *V17* and *V18*, with *V17* suffering more severe damage. This is because *V18*, as the main control switch, has a significantly higher drain-source current during operation than *V17,* which acts as a current-limiting transistor. In VDMOS devices, numerous electron-hole pairs are generated in the gate oxide, and when the current is large, a substantial number of electrons rapidly fill the traps, promoting a positive feedback effect on the electron-hole recombination rate, which reduces the damage and traps. The similar decrease in saturation drain current further suggests that the gate structure and epitaxial layer do not have a significant effect on displacement damage, consistent with simulation results from the literature [15,16].

**Figure 7:**
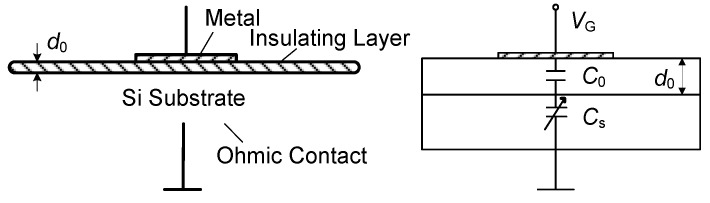
MIS structure and equivalent circuit.

**Figure 8:**
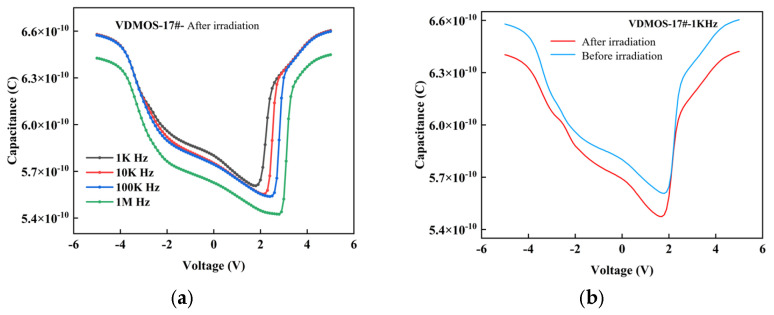

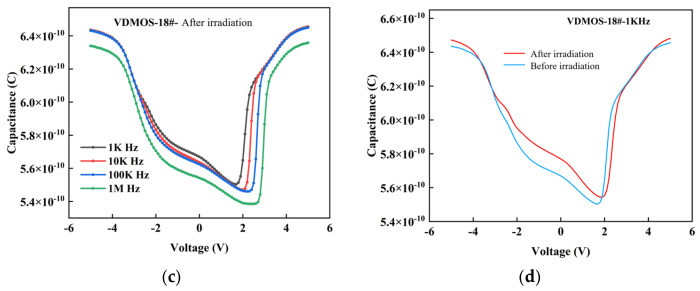
*C-V* characteristics of MIS structures post neutron irradiation: (**a**) Multi-frequency *C-V* characteristics of *V17* MIS structure, (**b**) Comparative output characteristics (pre-/post-irradiation) of *V17* MIS structure, (**c**) Multi-frequency *C-V* characteristics of *V18* MIS structure, (**d**) Comparative output characteristics (pre-/post-irradiation) of *V18* MIS structure.

As shown in Figure 7, the MIS structure and equivalent circuit of the MOSFET are illustrated. The MIS structure consists of metal, oxide (SiO_2_), and silicon substrate, resulting in a gate capacitance that includes the oxide capacitance, depletion layer capacitance, and interface state capacitance. The *C-V* curve reflects the characteristics of these capacitances as a function of gate voltage [17,18], and it is a key method for studying the semiconductor surface and interface. Figure 8 shows the *C-V* characteristic curves of the MIS structure after neutron irradiation.

In an ideal MIS structure, the capacitance is equivalent to the series combination of the insulating layer capacitance and the semiconductor space charge layer capacitance. The relationship is given by Equation (7), and the equivalent circuit is shown in Figure 7.

(7) $$C=1/(\frac{1}{C_{0}}+\frac{1}{C_{s}})$$

where *C* is the MIS structure capacitance, *C_0_* is the insulating layer capacitance, and *C_s_* is the semiconductor space charge layer capacitance.

From Figure 8, it is evident that the insulating layer capacitance of the VDMOS device remains largely unchanged after neutron irradiation, while the semiconductor space charge layer capacitance changes, indicating the generation of trap charges in this layer. The trap charge density in *V17* is higher than in *V18*.

**Figure 9:**
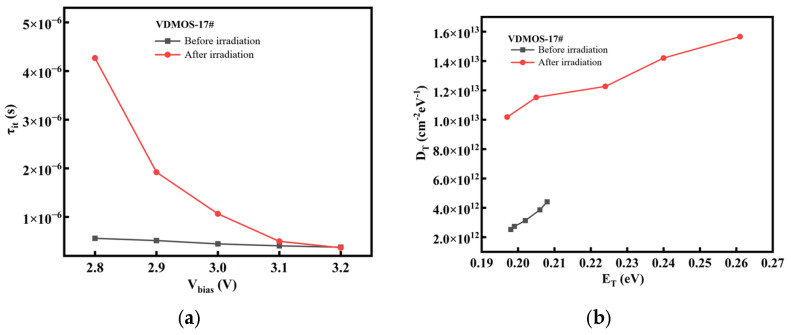

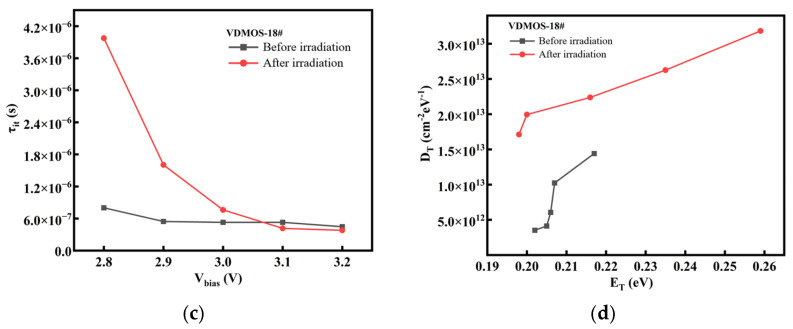
Comparison of Trap Time Constants and Concentrations Before and After Irradiation: (**a**,**b**) Characteristics of *V17*; (**c**,**d**) Characteristics of *V18*.

Post-irradiation, deep-level traps are formed in the VDMOS devices, as shown in Figure 9. The trap time constant increases by a factor of approximately four under low bias, meaning that once carriers are captured, their release is slower. This degrades the device’s response speed, reduces switching speed, and increases power loss. Neutron irradiation also elevates the trap density. The fast neutrons collide with silicon atoms, generating Frenkel defects that act as carrier traps. If a large number of such traps exist when the device is on, the number of free carriers decreases, leading to an increase in *R*_DS_(ON). This mechanistic explanation supports the observed degradation in electrical performance post-irradiation. Comparing the irradiation results of *V17* and *V18*, *V17* exhibits a higher concentration of radiation-induced defects and a longer trap time constant. This indicates that under cold standby conditions, devices like *V17* are more susceptible to severe degradation from neutron-induced displacement effects.

## 7. Conclusions

A DC/DC auxiliary power supply suitable for operation in a nuclear environment was designed to power chips such as PWM controllers or FPGAs that operate within a wide input voltage range. After neutron irradiation with a fluence of 3 × 10^13^ n/cm^2^, the auxiliary power supply voltage dropped to 8.9 V, but the chip continued to function normally. However, to maintain a stable supply voltage during irradiation, a low-dropout linear regulator (LDO) would need to be added in the post-regulation stage. The potential drawback of this modification is a reduction in efficiency. The changes in the electrical performance of key power VDMOS devices after neutron irradiation were analyzed. Specifically, the threshold voltages of the main power switch and current-limiting switch decreased from 5 V to 3.4 V and 3.2 V, and their on-resistances increased to 1.25 Ω and 1.38 Ω. This is attributed to the fact that during RCC operation, the drain-source current of the main power VDMOS transistor is larger than that of the current-limiting transistor. Neutron irradiation causes rapid recombination of traps, which significantly reduces displacement damage. By analyzing the performance degradation of the VDMOS after irradiation, we optimized the internal parameters of the RCC. This enables the system to withstand a higher neutron fluence and extend its operational duration.

The authors wish to acknowledge Professor Zhang Maolin from Xidian University for providing semiconductor parameter testing and discussing the results.

## Figures and Tables

**Table 1:** Electrical parameters of the DC-DC converter before neutron irradiation.

Input Voltage (V)	Input Current (A)	Output Voltage (V)	Voltage Variation (mV)	Load Variation (mV)	Efficiency
70	0.275	12.031	-	-	77.80%
120	0.163	12.043	12	-	76.50%
100 (Typical value)	0.191	12.038	-	-	78.20%
100	0.03 (No load)	12.036	-	2	-

**Table 2:** Electrical parameters of the DC-DC converter after neutron irradiation.

Input Voltage (V)	Input Current (A)	Output Voltage (V)	Voltage Variation (mV)	Load Variation (mV)	Efficiency
70	0.277	12.015	-	-	77.30%
120	0.164	12.031	16	-	76.10%
100 (Typical value)	0.194	12.017	-	-	77.50%
100	0.04 (No load)	12.005	-	12	-

**Table 3:** Electrical parameters of the RCC circuit before neutron irradiation.

Input Voltage (V)	Input Current (A)	Output Voltage (V)	Voltage Variation (mV)	Load Variation (mV)	Efficiency
70	0.049	12.142	-	-	58.3%
120	0.026	12.186	44	-	63.1%
100 (Typical value)	0.032	12.163	-	-	61.8%
100	0.005 (No load)	12.231	-	68	-

**Table 4:** Electrical parameters of the RCC circuit after neutron irradiation.

Input Voltage (V)	Input Current (A)	Output Voltage (V)	Voltage Variation (mV)	Load Variation (mV)	Efficiency
70	0.053	8.805	-	-	53.1%
120	0.028	9.151	346	-	58.3%
100 (Typical value)	0.036	8.981	-	-	56.3%
100	0.10 (No load)	9.183	-	202	-

**Table 5:** VDMOS Electrical Characteristics.

Parameters	Test Conditions	Minimum Value	Maximum Value
*V*_(BR)DSS_	*V*_Gs_ = 0 V, *I*_D_ = 1 mA	150 V	-
*V*_GS(th)_	*V*_Ds_ = *V*_Gs_, *I*_D_ = 1 mA	1.5 V	5 V
*I*_GSSF_	*V*_GS_ = 20 V, *V*_DS_ = 0 V	−200 nA	200 nA
*I*_GSSR_	*V*_GS_ = 20 V, *V*_DS_ = 0 V	-	-
*I*_DSS_	*V*_Gs_ = 0 V, *V*_Ds_ = 80 V	150 V	100 μA
*R*_DS(ON)_	*V*_Gs_ = 12 V, *I*_D_ = 5 A	1.5 V	460 mΩ

## Data Availability

Data are contained within the article.

## References

[1] Talukder A., Ifty M.R., Al Fahad A. (2025). Comprehensive review of GaN HEMTs: Architectures, recent developments, reliability concerns, challenges, and multifaceted applications. e-Prime—Adv. Electr. Eng. Electron. Energy.

[2] Pan S., Feng S., Li X., Feng Z., Lu X., Bai K., Zhang Y. (2024). Effects of 1 MeV electron radiation on the AlGaN/GaN high electron mobility transistors. J. Semicond..

[3] Yang J., Sun X., Yu X., Qin Z., Li X. (2020). Characteristics of displacement defects in PNP transistors caused by heavy ion irradiation. Nucl. Instrum. Methods Phys. Res. Sect. B Beam Interact. Mater. At..

[4] Ye E.-L., Lai Y.-F., Shen C.-X., Hou Y.-J., Nan H.-J. (2025). Simulation of displacement damage in Si & SiO_2_ caused by protons. Radiat. Phys. Chem..

[5] Zimmaro A., Zoppo V., Ferraro R., Danzeca S. (2025). Investigation of Radiation Effects on GaN HEMTs for Particle Accelerators. CERN Indico. https://indico.cern.ch/event/1500025/contributions/6323254/attachments/3004809/5296426/Investigation%20of%20Radiation%20Effects%20on%20GaN%20HEMTs%20for%20Particle%20Accelerators.pdf.

[6] Wang C., Bai X., Chen W., Yang S., Liu Y., Jin X., Ding L. (2015). Simulation of synergistic effects on lateral PNP bipolar transistors induced by neutron and gamma irradiation. Nucl. Instrum. Methods Phys. Res. Sect. A Accel. Spectrometers Detect. Assoc. Equip..

[7] Wu K., Lv X., Zou D., Lu Y., Li J., Zhao Y. (2022). Neutron flux effect in silicon-based bipolar junction transistors exposed to californium-252. Nucl. Instrum. Methods Phys. Res. Sect. A Accel. Spectrometers Detect. Assoc. Equip..

[8] Li Q., Lu J., Situ Z., Liu X., Zhang Y., Kong X., Li C. (2024). Study of irradiation damage in silicon at different scales. Mater. Today Commun..

[9] Rasel M.A.J., Stepanoff S.P., Wetherington M., Haque A., Wolfe D.E., Ren F., Pearton S. (2022). Thermo-mechanical aspects of gamma irradiation effects on GaN HEMTs. Appl. Phys. Lett..

[10] Hu P.-P., Xu L.-J., Zhang S.-X., Zhai P.-F., Lv L., Yan X.-Y., Li Z.-Z., Cao Y.-R., Zheng X.-F., Zeng J. (2025). Failure mechanisms of AlGaN/GaN HEMTs irradiated by high-energy heavy ions with and without bias. Nucl. Sci. Technol..

[11] Feng Z., Pan S., Feng S., Zheng X., Wang Y., Zhang B., Lu X. (2025). γ-irradiation induced trapping effects on off-state and on-state p-GaN Gate high-electron-mobility transistors. Mater. Sci. Semicond. Process..

[12] Ravindran R., Massoud A.M. (2025). An overview of wide and ultra wide bandgap semiconductors for next-generation power electronics applications. Microelectron. Eng..

[13] Zhang G., Dong S., Xin Q., Guo L., Wang X., Xin G., Qin N., Lan X., Guo C., Wang W. (2026). Transistor-level thermal management in wide and ultra-wide bandgap power semiconductor transistors: A review. Int. J. Therm. Sci..

[14] Li H., Guo H., Cao R., Tu H., Huang X., Xue Y., Zeng X. (2025). Study on electron and gamma irradiation effects and damage mechanism of GaN HEMT. Microelectron. Reliab..

[15] Baba T., Siddiqui N.A., Saidin N.B., Yusoff S.H.M., Sani S.F.B.A., Karim J.A., Hasbullah N.F. (2024). Radiation-induced degradation of silicon carbide MOSFETs–A review. Mater. Sci. Eng. B.

[16] Goud R.S.P., Akkanaboina M., Machiboyina S., Kumar K.R., Anjum A., Khan S.A., Prakash A.P.G., Pathak A.P., Rao S.V.S.N. (2024). A study on the gamma and swift heavy ion irradiation-induced effects on the electrical properties of TaOx-based MOS capacitors. Nucl. Instrum. Methods Phys. Res. Sect. B Beam Interact. Mater. At..

[17] Neamen D.A. (2012). Semiconductor Physics and Devices: Basic Principles.

[18] Streetman B.G., Banerjee S.K. (2015). Solid State Electronic Devices.

